# Diseases Admitted under the Care of the Physicians of the Westminster Hospital, from the 18th of Jan. to the 20th of Feb. 1800

**Published:** 1800-03

**Authors:** 


					20$ ? State of Difeafes in London.
Difeafes admitted under the care of the Phyjicians of the W'.JlminJier
Hofpital, from the 18 th of fan. to the zoth of Feb. 1800,
Fevers - - 14
PJeurify - 8
Hepatitis - - I
Sore Throat - 2
Amenorrhcea - - 4
Anafarca 4
Afcites 3
Afthma 2
Catarrh . 2
Cough - - - 17
Diarrhoea ' - "9
Dyfentery - - - 2
DyfpepGa * - - 1 2
Dyfpnoea 2
Dyfuria - i
Epilepfy I
Gaftrodynia - - 2.
Hzemoptoe - 2
Hooping Cough - 1
Hypochondriacs - 1
Hyileria - 2
Leucorrhoea - .-2
Lithiafis - - 1
Phthifis - 5
Pleurodynia 1
Rheumatifm - 8
Vertigo. . - - x
Vomiting - r ? 1
Worms - - - 2*

				

